# Effective photocatalytic degradation of amoxicillin using MIL-53(Al)/ZnO composite

**DOI:** 10.1007/s11356-022-20527-0

**Published:** 2022-05-11

**Authors:** Asmaa Fawzy, Hani Mahanna, Mohamed Mossad

**Affiliations:** Public Works Engineering Department, Faculty of Engineering, Mansoura University, Mansoura, 35516 Egypt

**Keywords:** MIL-53(Al)/ZnO, Amoxicillin, Photocatalysis, Response surface method, Potassium periodate

## Abstract

A promising hierarchical nanocomposite of MIL-53(Al)/ZnO was synthesized as a visible-light-driven photocatalyst to investigate the degradation of amoxicillin (AMX). MIL-53(Al)/ZnO ultrafine nanoparticles were obtained by preparing Zn-free MIL-53Al and employing it as a reactive template under hydrothermal and chemical conditions. The synthesized nanocomposite (MIL-53(Al)/ZnO) has a low content of Al > 1.5% with significantly different characterizations of the parent compounds elucidated by various analyses such as SEM, TEM, XRD, EDX, and UV–Vis. The effect of operational parameters (catalyst dose (0.2–1.0 g/L), solution pH (3–11), and initial AMX concentration (10–90 mg/L)) on the AMX removal efficiency was studied and optimized by the response surface methodology. A reasonable goodness-of-fit between the expected and experimental values was confirmed with correlation coefficient (*R*^2^) equal to 0.96. Under the optimal values, i.e., initial AMX concentration = 10 mg/L, solution pH ~ 4.5, and catalyst dose = 1.0 g/L, 100% AMX removal was achieved after reaction time = 60 min. The degradation mechanism and oxidation pathway were vigorously examined. The AMX degradation ratios slightly decreased after five consecutive cycles (from 78.19 to 62.05%), revealing the high reusability of MIL-53(Al)/ZnO. The AMX removal ratio was improved with enhancers in order ($${\mathrm{IO}}_{4}^{-}$$> H_2_O_2_ > S_2_O_8_^−2^). The results proved that 94.12 and 98.23% reduction of *COD* were obtained after 60 and 75 min, respectively. The amortization and operating costs were estimated at 3.3 $/m^3^ for a large-scale photocatalytic system.

**Supplementary Information:**

The online version contains supplementary material available at 10.1007/s11356-022-20527-0.

## Introduction

Water is one of the most important compounds necessary for life on Earth and human health. Recently, pharmaceuticals and personal care products have been observed in various ecosystems such as groundwater, surficial water, sewage effluents, and even potable drinking water, potentially causing toxic events (Abazari et al. 2018). These substances can reach the aquatic environment through various sources as pharmaceutical manufacturing industries, hospital effluents, and excretion from humans and livestock (Elmolla and Chaudhuri 2010). Antibiotics are one of various emerging pharmaceuticals being detected in many water systems (Chaba and Nomngongo 2019). Amoxicillin (AMX), as a member of the semi-synthetic β-lactam families of antibiotics used in human and veterinary medicines, is a drug widely used to treat bacterial infections such as the throat, ear, nose, skin, and lower respiratory tract (Chaba and Nomngongo 2019). According to previous reports, if 500 mg of AMX is administered orally in humans, excretion of 86 ± 8% in urine within 2 h of consumption results from its slow metabolism in the human body (Martins et al. 2009). Although AMX is sensitive to hydrolysis under pH conditions (Nägele and Moritz 2005), complete removal of AMX is complicated and can still be present in both urine and feces, along with its various hydrolyzed and metabolized by-products (Kanakaraju et al. 2015). Due to its chemical stability, high toxicity, and low biodegradation rate, this antibiotic causes alterations in the aquatic environment, which are subsequently grave for human health.

The application of physicochemical processes such as adsorption, coagulation, and filtration face many challenges that have arisen in previous studies. These drawbacks are high cost, low efficiency, and production of large volumes of sludge that requires further treatment or activation (Jorfi et al. 2018). Thus, it is crucial to find suitable alternative technologies for removing toxic and biologically persistent antibiotics in wastewater (Palma-Goyes et al. 2016). Recently, advanced oxidation processes (AOPs) have been progressively developed as a highly effective alternative for converting organic materials into simple mineral products (Trovó et al. 2011). The most common regarding the activation mode are Fenton reactions, ozonation, electrochemistry, photocatalysis, and ultrasonication. Among the AOPs from the point of view of global energy crises, photocatalysis is the most promising solution for converting photon energy into chemical energy in a sustainable way. Moreover, the photocatalysis has attracted the attention of the researchers for its applications in environmental cleanup due to its non-selectivity, high efficiency in removing recalcitrant pollutants, and effective reduction of toxicity (Dehghan et al. 2018). Nonetheless, it was noticed that the complete degradation of AMX attained in several studies required a long time (≥ 240 min) and reached a low removal ratio (≤ 40%) (Leong et al. 2015). Hence, the discovery of novel superior photocatalysts is needed to overcome these shortcomings.

Nano zinc oxide (ZnO) is one of the widely used semiconductors in photocatalytic applications, due to its various merits such as non-toxicity, eco-friendly properties, natural abundance, low price, photochemical stability, high surface area, and direct preparation methods (Ani et al. 2018). However, the limitation in harvesting visible light, rapid photo excited e^−^/h^+^ pair recombination, high bandgap (⁓3.3 eV), and the required excitation energy of 60 MV, are the inherent hindrances to obtain higher photocatalytic activity from nanostructures of ZnO. Moreover, another common limitation of ZnO is photocorrosion, which leads to a severe reduction in the photostability of ZnO during the recycling process under light irradiation (Han et al. 2014). The performance of such catalysts can be improved by optimization of micro and nanostructures (Xiao et al. 2019). Such a hierarchical structure is essential for the photocatalytic reaction at numerous levels. First, the absorption efficiency can be improved due to the scattering of the incident light on the surface of the multi-level structure. Second, charge-hole recombination may be reduced, and its presence on the surface of the photocatalyst can be enhanced. Finally, the hierarchical structure from the macro to micro level is a common target for achieving high surface area and thus increasing the number of active sites on the photocatalyst. Several recently synthesized approaches have been developed to prepare ZnO with hierarchical microstructures such as nanowires (Ko et al. 2016), nano flowers (Liu et al. 2016), and nanorods (Chen and Chuang 2012).

In a continuous effort, Zn-free metal–organic frameworks (MOF) are used as precursors to synthesize ZnO with a unique structure via the thermodynamic method. This latter is well known and available simply as hybrid coordination composites, which combine organic ligands with functional groups and metal centers (Xiao et al. 2019). Among MOFs, MIL-53Al (Al (OH) [O_2_C-C_6_H_4_-CO_2_] has attracted more attention due to its easy preparation, large surface area, and high porosity. In this work, MIL-53Al is converted by the reaction, at the same time providing a possible template, a potential source of other metal such as Al, and a possibly doping element of ZnO, not considered a Zn-source. In addition, MOF was present in a small amount compared to that of ZnO precursors.

In the present study, MIL-53(Al)/ZnO was synthesized and characterized. Then, we applied the novel photocatalyst in studying the degradation efficiency of AMX. Furthermore, the operating parameters were optimized with RSM to maximize the AMX removal. The degradation mechanism was investigated using radical quenchers. Five consecutive runs were carried out to assay the reusable capacity of MIL-53(Al)/ZnO. Some inorganic oxidizers such as KIO_4_, Na_2_S_2_O_8_, and H_2_O_2_ were employed to enhance the efficiency of AMX degradation. Also, the intermediate products were identified and discussed based on the mass spectroscopy (LC–MS/MS). A cost estimation study of a large-scale photocatalytic reactor including capital and running costs was also conducted.

## Materials and methods

### Materials

Amoxicillin and nano zinc oxide was purchased from Alfa Aesar (USA). Ethanol, terephthalic acid, aluminum nitrate nonahydrate, zinc acetate, potassium hydroxide, N, N-dimethylformamide (DMF), acetonitrile, monopotassium phosphate, orthophosphoric acid, potassium periodate, sodium persulfate, and hydrogen peroxide were supplied by Sigma-Aldrich. All chemical products were utilized directly without any modification.

### The photocatalyst preparation

According to a previously reported procedure for preparing MIL-53Al (Xiao et al. 2019). First of all, terephthalic acid (1.55 g, 9.36 mmol) was dissolved in (120 mL) of DMF. Second, Al (NO_3_)_3_·9H_2_O (2.36 g, 6.3 mmol) was stirred with the terephthalic acid solution for 30 min at 25 °C. Then, the mixture was transferred to a 200 mL autoclave and stored for 72 h at 220 °C. A white powder was produced after cooling the solution to 25 °C. The obtained product was washed three times with aqueous DMF and ethanol to remove impurities. Before obtaining the final product, the white powder was vacuum dried for 12 h at 50 °C. The final white powder product of 1.65 g of MIL-53Al with a yield of 42.01% was cooled to room temperature.

MIL-53(Al)/ZnO nanocrystals with a mass ratio of (1:6.23) were synthesized by a hydrothermal process using the prepared MIL-53Al as a reactive template with the following detailed procedures. First, the powder of MIL-53Al (0.104 g, 0.5 mmol) was slowly mixed in a beaker containing 0.2-M (40 mL, 8 mmol) zinc acetate solution with continuous stirring (400 rpm) for 30 min at 25 °C. Then, a 2-M potassium hydroxide solution (16 mL, 32 mmol) was gradually added to the mixture and stirred for 30 min. Then, the suspension was transferred to a 100 mL autoclave and reacted at 200 °C for 12 h. Next, the mixture was cooled to 25 °C and centrifugated at 8000 rpm for 15 min to separate the solid product from the liquid. The prepared catalyst was washed with anhydrous ethanol (50 mL) and then was dried at 50 °C for 24 h. The collected particles were ground in an agate mortar to get (0.65 g) a fine white powder of MIL-53(Al)/ZnO. Finally, the powder was calcined at 400 °C for 3 h. After calcination, the yield of the obtained sample of MIL-53(Al)/ZnO was above 99%. The intermediate reactions were summarized in the following Eqs. (1)–(2):

1 $${\mathrm{ZnC}}_{4}{\mathrm{H}}_{6}{\mathrm{O}}_{4}+2\mathrm{KOH}\to {\mathrm{K}}_{2}{\mathrm{C}}_{4}{\mathrm{H}}_{6}{\mathrm{O}}_{4}+\mathrm{Zn}{\left(\mathrm{OH}\right)}_{2}$$

2 $$\mathrm{Zn}{\left(\mathrm{OH}\right)}_{2}\rightarrow \mathrm{ZnO}+{\mathrm{H}}_{2}\mathrm{O}$$

### Experimental setup

In this study, the photoreactor comprises a 250-mL Pyrex beaker containing 250 mL of aqueous AMX solution. A metal halide lamp was used to illuminate the catalyst in all the experiments with a power of 400 W and a maximum wavelength of 510 nm. The distance between the light and the surface of the solution is ~ 11 cm. During the first 30 min of reaction, the reaction was performed in the dark to fulfill the adsorption phase. Then, the light was switched on to begin the photocatalytic process. The initial AMX concentration was changed in the range (10–90 mg/L), different catalyst doses (0.2–1.0 g/L) were added in a suspension state, and the pH of the solution was adjusted from 3 to 11 using 1 M NaOH and 1 M HCL to study their effect on the efficiency of AMX degradation. The AMX concentration was measured using HPLC (Agilent 1200 series, USA) with a C-18 Phenomenex reverse phase column. The mobile phase was a mixture of 0.05 M of a KH_2_PO_4_ buffer solution acidified with H_3_PO_4_ at pH = 3 and acetonitrile at the ratio 90 to 10% (V:V), respectively. During irradiation, sample aliquots were taken every 15 min for 1 h and filtered through 0.22-µm membrane filters before moving to the auto-sampler of HPLC. At a retention time of about 2.8 min, furnace temperature of 25 °C, flow rate of 1 mL/min, and injection volume of 10 µL, the peak of the detected signal of AMX was at a wavelength of 230 nm. The AMX limit of detection was determined using the detection limit (DL) method explained in Table S.1. The DL was determined to be 0.087 mg/L. The same suspended catalyst was used for five consecutive runs to examine the reusability and stability of MIL-53(Al)/ZnO. The transformation products were identified using tandem mass spectroscopy. The mobile phases A and B were acetonitrile and distilled water dissolved with 0.1% formic acid, respectively. The percentage of solution B increased from 10 to 90% in 10 min and remained constant for 3 min. The column temperature and the flow rate were 40 °C and 0.4 mL/min, respectively. The AMX degradation was also evaluated in the experiment by measuring the chemical oxygen demand using a *COD* analyzer, according to APHA (2005).

### Analytical methods

The point zero charge (PZC), at which the synthesized catalyst was neutrally charged in the reaction solution, was determined by the powder addition method as follows: a 0.2 g/L of MIL-53(Al)/ZnO was added to various mixtures of 1 M NaOH and 1 M HCL were used to adjust the pH of the solution to 2, 4, 6, 8, 10, and 12. After stirring them for 24 h at 25 °C, the final pH values were measured (Zhao et al. 2016). As depicted in Fig. S.1, the relationship between the initial and final pH values was plotted to estimate PZC.

The crystalline properties of the prepared catalyst were analyzed by an (XRD 600 Shimadzu, Japan) X-ray diffractometer (XRD) with Cu Kα radiation (*λ* = 1.54 A°). A transmission electron microscopy (TEM, JEOL, JEM-2100, Japan) equipped with selected area electron diffraction (SAED) was used to investigate the detailed morphological properties of the catalyst by taking high-resolution images. The surface morphology of the prepared catalyst was examined by scanning electron microscopy (SEM, Hitachi FESEM-4800) combined with energy-dispersive X-ray spectroscopy (EDX) to reveal the major chemical elements of MIL-53(Al)/ZnO. N_2_ adsorption–desorption analysis was carried out by the Belsorp-max automated apparatus (BEL Japan) to determine the specific surface area and the pore size distribution based on Brunauer–Emmett–Teller. A smart Omni-transmission Fourier-transform infrared spectroscopy (FT-IR) was used to identify the functional groups of the prepared catalyst. Bandgap evaluation was performed by the Kubelka–Munk function, based on data from (V-630 UV–Vis Spectrophotometer, Jasco). Photo-luminescence emission spectra (PL, Perkin Elmer, LS45, spectrofluorometer) were recorded within an excitation wavelength in the range of 200 to 800 nm to determine the separation of electrons and holes.

### Design of experiments by RSM

The RSM was employed to investigate and optimize the operating parameters (pH, catalyst dose, and the initial AMX concentration) on the efficiency of AMX degradation. The different levels and the corresponding ranges of the operational conditions by central composite design (CCD) are in Table 1.

**Table 1:** CCD levels and ranges of operating conditions for AMX degradation

Independent variables	Units	Levels				
		− 2	− 1	0	1	2
pH	-	3	5	7	9	11
Catalyst dose	g/L	0.2	0.4	0.6	0.8	1
AMX concentration	mg/L	10	30	50	70	90

In this work, a set of 18 runs consisting of 6-axial, 8-factorial, and 4-central points was performed to achieve a good CCD design for optimizing the operating conditions and to obtain an excellent model representing the interaction between the operational parameters. The relationship between operating parameters and the AMX degradation efficiency can be expressed by a quadratic equation as displayed in Eq. (3) (Hou et al. 2012):

3 $$Y\left(\%\right)=\beta_0+\sum\nolimits_{\mathrm i=1}^{\mathrm n}{\mathrm\beta}_1{\mathrm X}_{\mathrm i}+{\textstyle\sum_{\mathrm i=1}^{\mathrm n}}{\mathrm\beta}_2\mathrm X_{\mathrm i}^2+{\textstyle\sum_{1<\mathrm i<\mathrm j}^{\mathrm n}}\;{\mathrm\beta}_{\mathrm{ij}}{\mathrm X}_{\mathrm i}{\mathrm X}_{\mathrm j}+\mathrm\varepsilon$$

where *Y* (%) is the percentage of AMX removal after 60 min of reaction time;${X}_{i}$ and ${X}_{j}$ are the independent variables; *β*_0_ is the intercept coefficient of the model; *β*_1_, *β*_2_, and *β*_*ij*_ are linear and quadratic coefficients; ***ε*** is the residual term; and *n* is the number of dependent variables. Using a multiple regression model, the coefficients of the quadratic equation were determined by fitting the experimental data to the response. The regression analysis was conducted using Minitab $\copyright$ 19 software. Furthermore, the analysis of variance (ANOVA) was employed to check the degree of fitness and the significance of the model, as represented by correlation coefficient *R*^2^, *F*, and *P*-values (Shayegan et al. 2013). The optimum values of the operating parameters were obtained according to a desired function to maximize the efficiency of AMX degradation as a target after an irradiation time of 60 min.

## Results and discussion

### Characteristics of MIL-53(Al)/ZnO

**Fig. 1:**
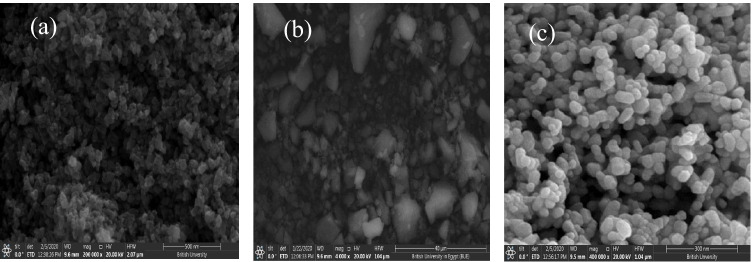
SEM images of (**a**) the synthesized photo catalyst MIL-53(Al)/ZnO;(**b**) MIL-53Al; (**c**) ZnO

**Fig. 2:**
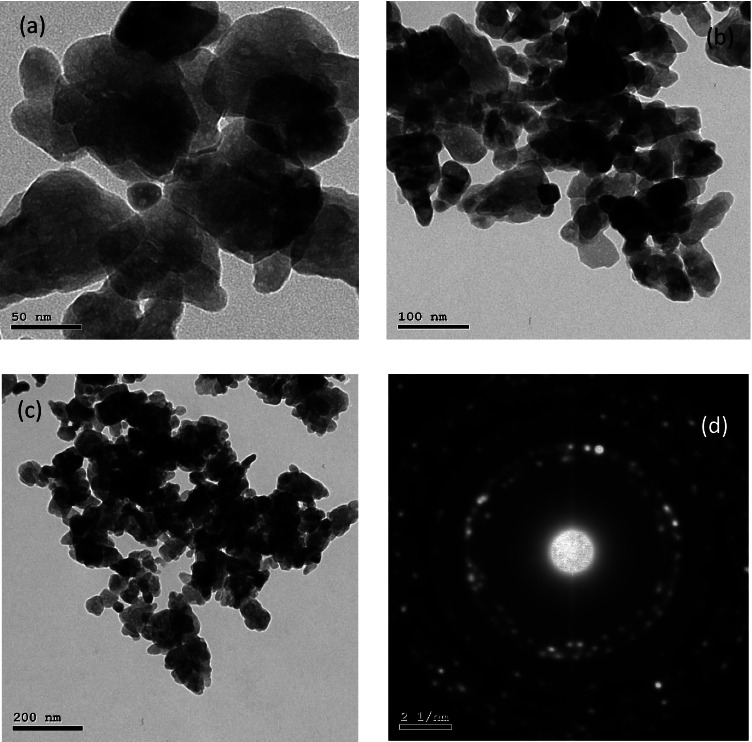
**a**–**c** TEM images, **d** SAED image of MIL-53(Al)/ZnO

The photocatalytic process is ruled by the morphological properties of the catalyst, such as the active surface and porosity. Small sizes achieve a high surface-to-mass ratio; subsequently, the short path the electrons have to take to reach the solid/solution interface led to higher efficiency. Thus, our target was to synthesize semi-conductors in nanocrystal structures where the number of incident photons increases. The SEM micrographs in Fig. 1 depict the morphology of MIL-53(Al)/ZnO, MIL-53Al, and pure ZnO. MIL-53(Al)/ZnO appears with a flake-like morphology with more ultrafine nanosheets in the sample, as shown in Fig. 1a. The SEM image confirmed the presence of a small cluster of particles in different sites as a result of sintering of MIL-53Al and ZnO crystals through the calcination process. On the other hand, MIL-53Al appears in Fig. 1b as sub micrometric particles (30–100 nm) with a morphological surface of oval and flat cubes with acute or round corners. Figure 1c exhibits the morphology of pure ZnO with a six-square prism structure (Xiao et al. 2019). TEM images represented in Fig. 2a–c revealed the growth of MIL-53(Al)/ZnO particles with a size range of 10 to 200 nm. The high-resolution TEM of MIL-53(Al)/ZnO displays the cohesion between MIL-53Al and ZnO with lattice spacing of each element in the synthesized catalyst, confirming the successful interaction between ZnO and MIL-53Al. Likewise, the SAED pattern in Fig. 2d indicates that the MIL-53(Al)/ZnO is polycrystalline material. In Fig. S.2 (a) and Table S.2, EDX analysis of MIL-53(Al)/ZnO calcined at 400 °C affirms the presence of Zn, O and low quantity of Al. The molar ratio of Al/Zn was 1/16; this low molar ratio was selected to display the influence of MOFs in low quantity on ZnO synthesis. Hence, the Al from MIL-53Al was evidently integrated into the MOF-templated ZnO sample.

**Fig. 3:**
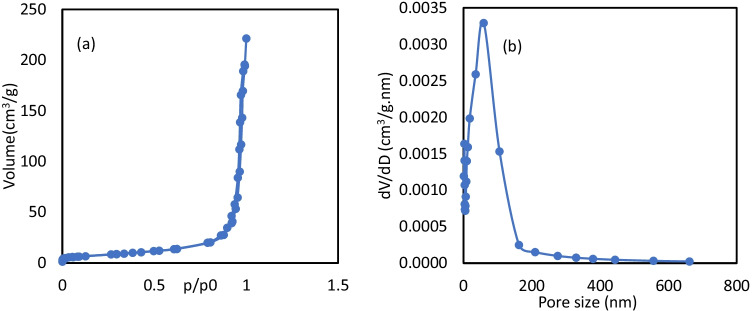
**a** Adsorption–desorption isotherm; **b** pore size distribution of MIL-53(Al)/ZnO

Figure 3a displays the isotherm of nitrogen-adsorption and desorption of MIL-53(Al)/ZnO. The *S*_BET_ was calculated for MIL-53(Al)/ZnO (27.45 m^2^/g), according to the BET method, indicating that the surface area of the catalyst prepared by compositing ZnO with MIL-53Al was smaller than the parent compounds due to the growth and the agglomeration of nanograins after the calcination process, as the measured specific surface area of MIL-53Al and ZnO were 776.8 and 28.39 m^2^/g, respectively. The relatively high specific surface area also leads to an increase in active sites at the photocatalyst interface. Figure 3b shows the pore size distribution of the photocatalyst determined by the BJH method. The pore size of MIL-53(Al)/ZnO was larger than that of ZnO and MIL-53Al, indicating a collapse of porous structure, causing larger mesopores (Xiao et al. 2019). The wide pore size distribution confirms the composition of the hierarchical structure and implies the presence of mesopore in the sample. In addition, the mesopore structure is desirable to reduce mass transfer and light-harvesting during photocatalysis. The absence of an adsorption limit at high *P*/*P*_0_ affirms the existence of macropores in the catalyst structure (Zhou et al. 2009). The wide pore size distribution allows the availability of contaminants to/from active sites on the surface of the photocatalyst. It also enhances the potential for reuse and regeneration of semiconductors (Li et al. 2016). Therefore, the method of preparing MIL-53(Al)/ZnO seems to have an optimistic effect on its morphological characteristics.

**Fig. 4:**
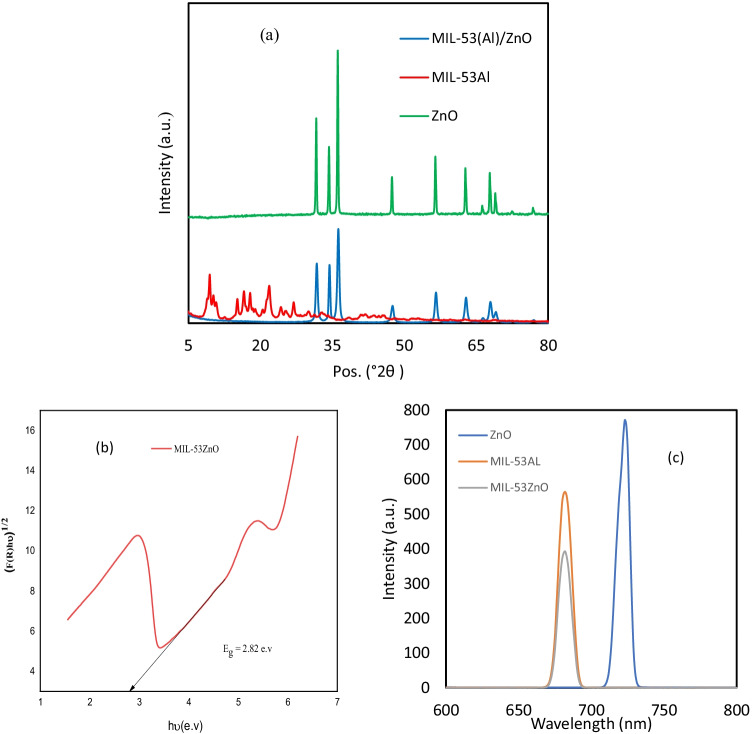
**a** XRD patterns of ZnO, MIL-53Al, MIL-53(Al)/ZnO; **b** determination of band gap of MIL-53(Al)/ZnO, **c** PL spectra

The ruling factor of the photocatalytic process is the semiconductor due to its dual roles of photo acceptor and reservoir of electrons. Figure 4a illustrates the XRD patterns of ZnO, MIL-53Al, and MIL-53(Al)/ZnO synthesized with MIL-53Al as a reactive template. All diffractograms exhibit the typical diffraction peaks at 2θ = 31.746°, 34.407°, 36.2462°, 47.5489°, 56.5663°, 62.8787°, 66.331°, 67.9447°, 69.0917°, 72.5716°, and 77.0267° indexed to reflections of ZnO (JCPDS36-1451) (Xiao et al. 2019). There are no major differences between the two patterns. The interaction of Al ions with ZnO resulted in small shifts of ZnO peaks recorded in the MIL-53(Al)/ZnO pattern. The structure of the catalyst seems like a hexagonal wurtzite in the XRD analysis. Also, the MOF structure was destroyed and transformed while the MIL-53(Al)/ZnO was generated. Furthermore, the unique diffraction peaks of any Al-containing oxides phases were absent in the spectra due to the low amount of Al in MIL-53(Al)/ZnO.

Figure S.2 (b) explains the FT-IR spectra. In addition, the stretching and bending vibration bonds are recorded and identified in Table 2. The results showed that all the functional groups ascribed to bare ZnO are present in the synthesized MIL-53(Al)/ZnO structure. There is no clear band ascribed to the stretching vibration mode of Al-O, confirming that MIL-53Al was only applied as a reactive template to produce ZnO nanocrystals.

**Table 2:** The identification of the catalyst functional groups

The stretching vibration bond	The band (Cm^−1^)	Reference
Zn–O	459.988	Largani and Pasha (2017)
O–H	3500	Mahanna and Samy (2020)
H–O–H	1630	Younes et al. (2019)
C–H	2920.287	El-Bendary et al. (2021)
Al–O	No clear band	Guha Ray et al. (2020)

The bandgap of a photocatalyst is a key optical factor that exhibits the formation of photo-excited electrons and holes. The optical bandgap of the prepared catalyst was calculated by the UV–Vis reflectance mode with the Kubelka–Munk relationship. The relationship between the photon energy (*hʋ*) and (*F*(*R*) *hʋ*)^1/2^ was used to calculate the bandgap of MIL-53(Al)/ZnO by extending the linear segment to intersect with the abscissa, as represented in Fig. 4b. The value of the bandgap is equal to the measure of the intercept with the x-axis (Samy et al. 2020a). The function of reflectance F(R) is illustrated in Eq. (4).

4 $$F\left(R\right)=\frac{{(1-\mathrm R)}^2}{2\mathrm R}$$

where *R* is the reflectance. The estimated bandgap for MIL-53(Al)/ZnO is 2.82 eV. The lower bandgap assures higher photocatalytic activity to remove resistant contaminants under various light sources such as metal halide lamps and solar light. Furthermore, the obtained bandgap approves that the compositing of pure ZnO with MIL-53Al resulted in the reduction of the bandgap of ZnO, as the reported ZnO bandgap is ~ 3.3 eV (Camaratta et al. 2015). MIL-53(Al)/ZnO has an effective photoreactivity in the visible light region, attributing to the prominent optical property. As described in Fig. 4c, photoluminescence spectra (PL) were carried out to determine the separation and recombination of the generated electrons and holes. The PL emission resulted from the recombination of photoinduced electrons and hales. The order of the PL intensity for MIL-53(Al)/ZnO, ZnO, and MIL-53Al was ZnO > MIL-53Al > MIL-53(Al)/ZnO. The lower PL intensity of MIL-53(Al)/ZnO indicates improved separation of electrons and holes (Khan et al. 2019).

### Statistical analysis and analysis of variance

The efficiency of AMX removal was investigated by response surface contour plots and a quadratic model. The detailed experimental data for measured and predicted removal ratios of AMX after a photocatalytic reaction time of 60 min are listed in Table S.3. The relationship between the independent parameters and the efficiency of AMX degradation is expressed by a quadratic response function Eq. (5):

5 $$Y \left(\mathrm{\%}\right) = 86.2 + 0.27 {X}_{1} + 42.5 {X}_{2} + 0.248 {X}_{3} - 0.473 {X}_{1}^{2}- 18.4 {X}_{2}^{2} - 0.00475 {X}_{3}^{2}- 1.04 {X}_{1}{X}_{2} - 0.0103 {X}_{1}{X}_{3} + 0.110 {X}_{2}{X}_{3}$$

where *Y* (%) is the efficiency of AMX degradation after 60 min; *X*_1_ is the pH of the solution; *X*_2_ is the dose of the photocatalyst in g/L; and *X*_3_ is the AMX initial concentration in mg/L.

The analysis of variance (ANOVA) emphasizes the significance and adequacy of the model. The high *F*-statistics and small *P*-values reveal the signature of the model exposed in Table S.4. The expected *R*^2^ and the adjusted *R*^2^ values are (95.69%) and (90.85%), respectively. These values, close to 1, ensure the relevance of this mode, which has high authenticity for the regression of data and can accurately represent the actual value. Besides, the low variance between the predicted and measured removal efficiency implies the model efficiency. Judging from the *F*-values of variables, the order of operating parameters according to their influence on the AMX degradation performance was pH of the solution > initial concentration of AMX > photocatalyst dose.

### Effect of the operational parameters on the degradation of AMX

**Fig. 5:**
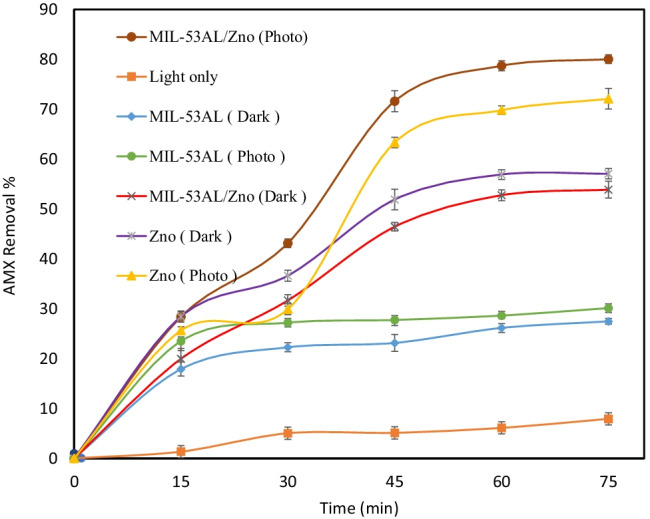
Degradation of AMX under different conditions, initial AMX conc = 50 mg/L, pH = 7, catalyst dose = 0.6 g/L

The removal of AMX studied under various conditions is shown in Fig. 5. A low degradation of 6.11% occurred after 60-min irradiation time, and this low removal ratio was ascribed to the poor light absorption of AMX (Dimitrakopoulou et al. 2012; Kanakaraju et al. 2015). So, the results assured that direct photolysis only marginally contributed to the decomposition of AMX. Higher removal rates were attained using photocatalysts in the presence of light. An 81.02% of AMX was removed after 60 min by MIL-53(Al)/ZnO compared to 69.81% and 28.63% using ZnO and MIL-53Al in the presence of light, respectively. This result indicated that the incorporation of pure ZnO with MIL-53Al decreased the catalyst bandgap and the charge recombination rate. However, the removal of AMX in the absence of light was higher using ZnO than using MIL-5(Al)/ZnO by around 4%, because ZnO has a higher surface area equal to 28 m^2^/g. But the photodegradation was better than the adsorption process and MIL-53(Al)/ZnO was confirmed being an efficient semiconductor. Therefore, MIL-53(Al)/ZnO was applied in all the following experiments in the presence of light due to its superiority.

**Fig. 6:**
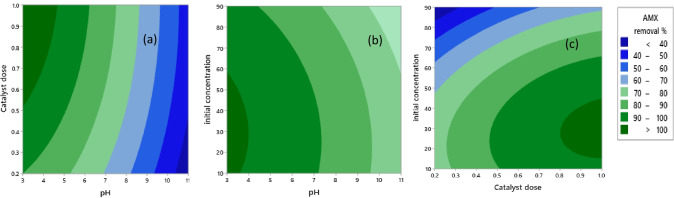
**a**–**c** contour plots represent the output of RSM for the effect of operating conditions on the AMX removal

The contour plots in Fig. 6a–c elucidate the influence of the catalyst dose, the initial AMX concentration, and the solution pH on the AMX removal efficiency. The optimum catalyst dose depends on the initial AMX concentration. Thus, it is necessary from a mechanistic and practical perspective to investigate the effect of the initial AMX concentration on the photocatalytic reaction rate. Increasing the initial AMX concentration above the optimum value (10 mg/L) reduced the removal of AMX due to further depletion of the active sites through the accumulation of AMX particles on the surface of the photocatalyst. Furthermore, at high initial AMX concentrations, the generated reactive oxidant species (ROS) are insufficient to complete a prolonged reaction with the organic pollutants causing the impediment to the desired efficiency. Thereby, a longer reaction period may be needed to reach the desired performance (Gar Alalm et al. 2018).

The higher photolysis efficiency was improved by increasing the doses of the catalyst, which was attributed to the production of more active sites on the surface of the catalyst up to the optimum value (1.0 g/L). Moreover, the increase in active sites that can absorb photons, resulting in greater absorption of AMX molecules leading to higher generation of ${\mathrm{OH}}^{-.}$, ${h}^{+}$, ${e}^{-1}$, and ${\mathrm{O}}^{-2.}$ and direct elimination of pollutants (Mirzaei et al. 2016). Nevertheless, the efficiency of AMX removal was slightly increased from 0.8 to 1.0 g/L, with the percentage removal efficiency equal to 85.67 and 88.93%, respectively. This result is consistent with the regression equation Eq. (5). Exceeding the optimum catalyst dose leads to a reduction in the removal efficiency. This increase may hinder the light from effectively penetrating and reaching the active sites of the photocatalyst due to the high turbidity of the solution (Neppolian et al. 2002).

The initial pH plays an important operational factor for adsorption and photoactivity. It administrates the ionization of the oxidant and the separation of active sites. So, there are two sets of knowledge. First, the point PZC was evaluated to verify the effect of pH on the surface charge of the semiconductor, and secondly, the optimal pH for the decomposition of AMX was determined.

As described in Fig. S.1, the PZC point for MIL-53(Al)/ZnO is ~ 8.4. Thus, the surface charge of MIL-53(Al)/ZnO was positive at solution pH less than 8.4 and vice versa. On the other hand, AMX shows different ionization states due to different ionizable functional groups, carboxyl (pKa_1_ = 2.69), amine (pKa_2_ = 7.49), and phenol (pKa_3_ = 9.63) (Serna-Galvis et al. 2016). As a result, the removal efficiency of AMX decreased at higher pH due to the electrostatic repulsion forces between the catalyst and the AMX particles. Whereas, in acidic conditions, the forces between the surface of the catalyst and the AMX particles were electrostatic attraction forces that led to an increase in the photolysis efficiency of AMX. As shown in Table 3, the optimum pH value for AMX removal is ~ 4.5 obtained from Minitab 19 statistical software, which is located between the pK_a1_ of AMX (2.69) and the PZC of MIL-53(Al)/ZnO.

**Table 3:** Optimal values of operational parameters and comparison of expected and experimental values of AMX degradation under the optimal conditions

Parameter	Optimum values
Photo catalyst dose (g/L)	1
pH of solution	4.48
AMX concentration (mg/L)	10
Enhancer dose KIO_4_ (mM)	2
Expected AMX removal (%) after 60 min	100%
Experimental AMX removal (%) after 60 min	100%

As listed in Table S.5, a comparison between the efficiency of AMX degradation in previous studies using different photocatalysts with the efficiency of AMX degradation in our work has been reported. Interestingly, a high level of degradation was achieved in a relatively short time with MIL-53/ZnO in this study.

### Photo catalysis mechanism 

**Fig. 7:**
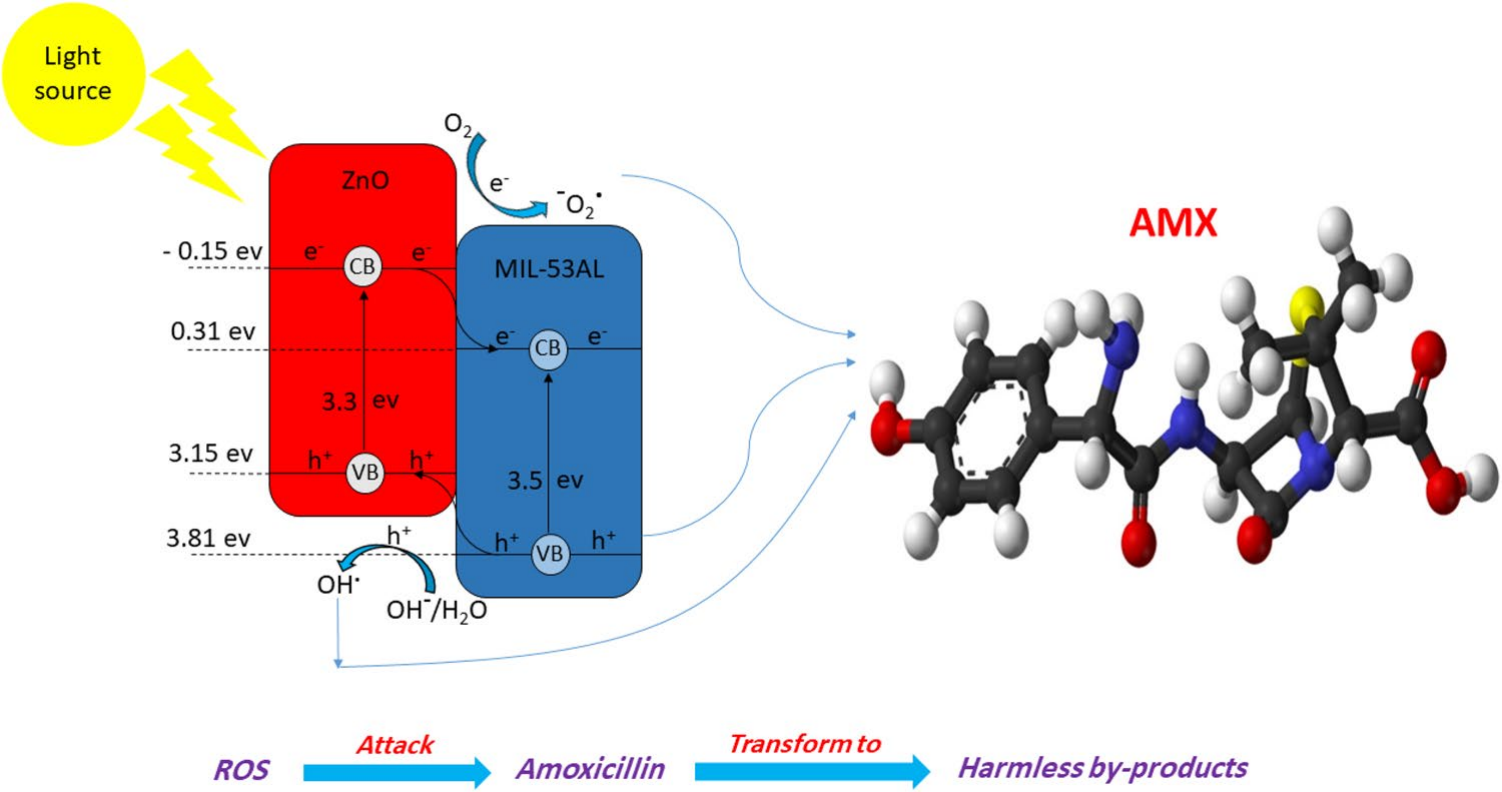
Illustration of charge transfer between ZnO and MIL-53Al and the generated ROS

Figure 7 and Table S.6 illustrate the mechanism of generation of ROS through the interaction between ZnO and MIL-53Al. A convenient light source illuminates the semiconductor by a higher energy than its excited-bandgap electrons, thus electrons move from the valence to the conduction band, leaving holes in the valence band and then generating electron/hole pairs. The conduction and valence band potential of a semiconductor can be calculated by the following Eqs. (6)–(7) (Mirzaei et al. 2018; Zhao et al. 2020):

6 $${E}_{\mathrm{VB}}=X-{E}^{e}+0.5{E}_{g}$$

7 $${E}_{\mathrm{CB}}={E}_{\mathrm{VB}}-{E}_{g}$$

where *E*_VB_ and *E*_CB_ are the potentials of valence and conduction bands, respectively; *X* is the geometric mean of the absolute electronegativity of the constituent atoms on the Pearson scale (PAE), (Samy et al. 2020b); *E*_g_ is the catalyst bandgap (Srikant and Clarke 1998), and *E*^e^ is the energy of electrons on hydrogen scale (~ 4.5 eV) (Grinnell and Samokhvalov 2018). Therefore, during the photocatalysis process, photons excite MIL-53(Al)/ZnO, then the electrons begin to move from the valence band (VB) to the conduction band (CB) and leave holes in the valence band (VB). The electrons react with dissolved oxygen to produce ${\mathrm{O}}^{-2.}$ radicals. The holes in VB react with H_2_O or ${\mathrm{OH}}^{-}$ and generate ${\mathrm{OH}}^{-.}$ radicals, as an important oxidant radical, which attack or absorb the organic molecules near to the active sites on the surface of the photocatalyst. In addition, the holes and the superoxide radicals (${\mathrm{O}}^{-2.}$) contribute to the increased oxidation of organic matter. It is reported that the electron–hole pair recombination rate is effectively reduced by charge transfer between semiconductors, which extends the lifetime of the formed charge carriers (Kumar et al. 2018). The decrease in PL emission intensity in the current work illustrated in Fig. 4c confirms this approach.

**Fig. 8:**
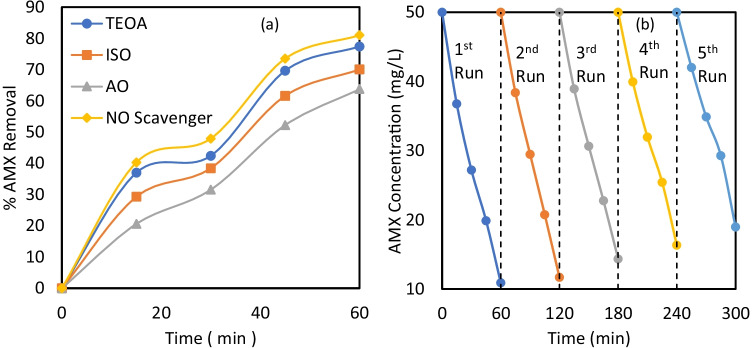
**a** Effect of different types of scavengers on the removal efficiency of AMX, **b** degradation efficiencies of AMX in 5 repetitive runs at pH = 7.0, catalyst dose = 0.6 g/L, initial AMX concentration = 50 mg/L, irradiation time = 60 min

Isopropanol (ISO), triethanolamine (TEOA), and ammonium oxalate (AO) were employed as quenchers of hydroxyl, hole, and superoxide radicals, respectively to quantify the participation of each ROS in the photocatalytic reaction (Molla et al. 2017). The degradation efficiency of AMX without scavengers was 81.02%, while the degradation efficiencies were 70.06%, 77.43%, and 63.75% with 1 mM of ISO, TEOA, and AO, respectively, as exhibited in Fig. 8a. The ${\mathrm{O}}^{-2.}$ radicals had effectively contributed to the degradation process, as depicted by the inactivation experiments. All types of reactive oxidants contributed to the photocatalytic decomposition process, confirming the oxidation mechanism.

### The reusability and performance of MIL-53(AL)/ZnO photocatalyst 

The catalyst-reuse performance was investigated using the same catalyst powder for five successive cycles. As elucidated in Fig. 8b, the photo-degradation efficiency of the suspended catalyst MIL-53(Al)/ZnO was 78.19%, 76.6%, 71.29%, 67.33%, and 62.05% in the five runs, respectively. The last cycles may require additional reaction time to achieve the desired decomposition. The gradual decrease in degradation efficiency at each subsequent run may be ascribed to the loss of catalyst molecules after long-run times, leaching of zinc ions into the solution, and accumulation and adsorption of AMX particles and their transformers on the active sites on the catalyst surface in each run. Nonetheless, the appropriate removal of AMX in the five cycles ensured the stable production of ROS on MIL-53(Al)/ZnO after long periods of light irradiation and extended reuse potential.

### Effect of inorganic oxidants

The electron–hole recombination in heterogeneous photocatalytic processes is a major energy-wasting step. An effective effort was made to reduce the electron–hole recombination by adding permanent oxidizing agents in the reaction. For this target, different optimal-state runs were performed to estimate the effect of different enhancers such as (potassium periodate (KIO_4_), sodium peroxydisulfate (Na_2_S_2_O_8_), and hydrogen peroxide (H_2_O_2_), on the AMX photocatalytic degradation. As demonstrated in Fig. S.3, the AMX photocatalytic removal rate was significantly increased to 75.43%, 80.87%, 90.48%, 100%, and 100% in the following conditions respectively, without enhancer (catalyst dose = 0.2 g/L), Na_2_S_2_O_8_ (catalyst dose = 0.2 g/L), H_2_O_2_ (catalyst dose = 0.2 g/L), without enhancer (catalyst dose = 1.0 g/L), and KIO_4_ (catalyst dose = 0.2 g/L) after reaction time of 60 min. Among all performed conditions, KIO_4_ achieved the maximum degradation efficiency, represented by ${\mathrm{IO}}_{4}^{-.}$, which was the strongest oxidizing agent in the AMX photocatalytic degradation. Also, the result achieved using KIO_4_ with a low catalyst dose is the same as when using a high catalyst dose, but without enhancers, which saves in economic terms. The significant increase in the removal efficiency of AMX by${\mathrm{IO}}_{4}^{-}$, can be attributed to the efficient capture of the electrons generated on the surface of the photocatalyst, as presented in (Eq. (8)) (Khataee et al. 2015). Furthermore, ${\mathrm{IO}}_{4}^{-}$ possesses a higher oxidation efficiency in the photocatalysis process, compared to peroxydisulfate and hydrogen peroxide (Khataee et al. 2016).

For H_2_O_2_, the improvement of the photocatalytic degradation of AMX is mainly due to the rate of excess formation of ${\mathrm{OH}}^{-.}$ radicals through the decomposition of hydrogen peroxide molecules by photocatalysis, according to Eq. (9) (Chen et al. 2014). The reduction in the conduction band effectively decomposed H_2_O_2_ to generate ${\mathrm{OH}}^{-.}$ radicals, as displayed in Eq. (10) (Dehghan et al. 2018).

Also, the photocatalytic degradation of AMX is positively affected by the decomposition of S_2_O_8_^−−2^ into ${\mathrm{SO}}_{4}^{-.}$ radicals via illumination as described in Eq. (11) (Jonidi Jafari et al. 2017). Moreover, ${\mathrm{SO}}_{4}^{-.}$ radicals react with H_2_O molecules to produce hydroxyl radicals, as shown in Eq. (12). However, S_2_O_8_^−2^ molecules can perform as a quencher for ${\mathrm{OH}}^{-.}$ radicals by converting them to lower oxidizing agents (S_2_O_8_^−.^), which leads to a slight decrease in the degradation efficiency, based on Eq. (13) (Dehghan et al. 2018). S_2_O_8_^−2^ has a very low degradation efficiency during the first 30 min (adsorption time) because persulfate anions can be activated by UV, US irradiation, heat energy, electron transfer of transition metal ions, or activated carbons. Persulfate anions can be converted to sulfate radicals, as illustrated in Eqs. (14) and (11) (Chen et al. 2014). In addition, this decrease in the efficiency of AMX removal at the time of adsorption may be related to the accumulation of persulfate molecules on the surface of the catalyst.

8 $${\mathrm{IO}}_{4}^{-}+8e+{8\mathrm{H}}^{+}\to {\mathrm{I}}^{-}+4{\mathrm{H}}_{2}\mathrm{O}$$

9 $${\mathrm{H}}_{2}{\mathrm{O}}_{2} +\mathrm{ UV }\to 2{\mathrm{OH}}^{-.}$$

10 $${\mathrm H}_2{\mathrm O}_2+\mathrm e^-\rightarrow\mathrm{OH}^{-.}+\mathrm{OH}^-$$

11 $${\mathrm{S}}_{2}{\mathrm{O}}_{8}^{-2}+\mathrm{heat}/\mathrm{UV}/\mathrm{US}\to 2{\mathrm{S}}_{4}^{-}$$

12 $${\mathrm{S}}_{2}{\mathrm{O}}_{8}^{-2}+{\mathrm{H}}_{2}\mathrm{O}\to {\mathrm{H}}^{+}+{\mathrm{OH}}^{-}+{\mathrm{SO}}_{4}^{-2}$$

13 $${\mathrm{S}}_{2}{\mathrm{O}}_{8}^{-2}+{\mathrm{OH}}^{-}\to {\mathrm{OH}}^{-}+{\mathrm{S}}_{2}{\mathrm{O}}_{8}^{-}$$

14 $${\mathrm{S}}_{2}{\mathrm{O}}_{8}^{-2}+{\mathrm{Me}}^{+\mathrm{n}}\to {\mathrm{SO}}_{4}^{-}+{\mathrm{SO}}_{4}^{-2}+{\mathrm{Me}}^{+\left(\mathrm{n}+1\right)}$$

### *COD* removal

The AMX degradation efficiency was also computed based on the chemical oxygen demand (*COD*) analysis via Eq. (15):

15 $$\mathrm{AMX}\;\mathrm{removal}\;\left(\%\right)=(1-\frac{{\mathrm{COD}}_{\mathrm{time}}}{{\mathrm{COD}}_{\mathrm{initial}}})\times100$$

**Fig. 9:**
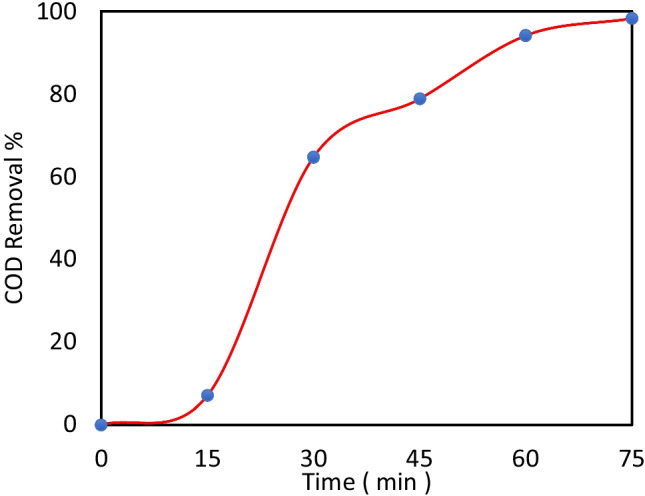
*COD* removal of AMX at initial conc. of AMX = 10 mg/L, catalyst dose = 0.2 g/L, pH = 4.5, and KIO_4_ dose = 2 mM

The change in *COD* during the photocatalytic process under the optimal operating parameters is displayed in Fig. 9, considering the change in *COD* as an indication of treatment. The *COD* removal was 7.1%, 64.71%, 78.82%, 94.12%, and 98.24% after 15, 30, 45, 60, and 75 min of reaction, respectively. At the early stages of the reaction, a slight removal of *COD* was observed due to the generation of intermediates in organic forms that also contain carbon. Further degradation occurred in the intermediate organic matter resulting in a significant decrease in *COD* in the later stages. This result confirms the promising feasibility of the MIL-53(Al)/ZnO compound for industrial wastewater treatment.

### Proposed degradation pathway of AMX

**Fig. 10:**
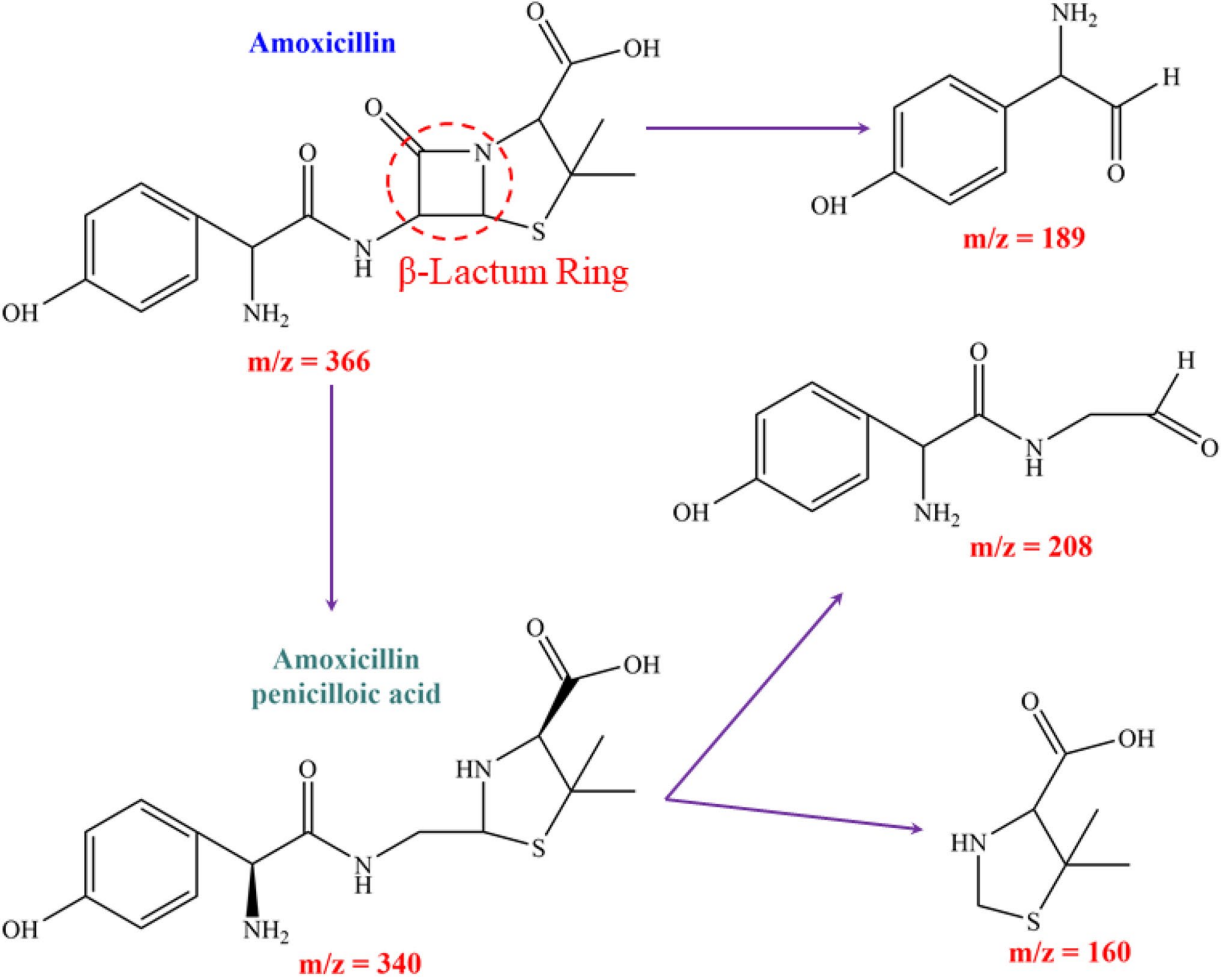
The suggested degradation pathway and identification of AMX by-products

The intermediate conversion products of AMX were determined by LC–MS/MS analysis of samples taken during the reaction. Hydroxyl radicals are generated as oxidizers during the photocatalysis process. Herein, as shown in Fig. S.4, AMX with a peak at *m*/*z* of 366 g/mol degraded by the opening β-lactum ring, lossing the amine group, and separating the bond between the hexagonal and pentagonal rings to produce amoxicillin penicilloic acid with *m*/*z* of 340 and another fragment at *m*/*z* = 189 (Jalali et al. 2021). the parent pollutant (AMX) was observed in the early-stage samples. The transformers with *m*/*z* of 208 and 160 were generated due to the oxidization of the methyl group. On the other hand, the transformation products at *m*/*z* of 223 and 206 were formed. These compounds are the fragments of ampicillin mixed with amoxicillin in the sample (Arsand et al. 2018). Moreover, the products with *m*/*z* of 391, 214, 195, and 158 were contaminants in the water of LC-mobile phase. The continuous oxidation of these intermediates was finally converted by photochemically generated reactive oxidizing species into simple by products such as, water, carbon dioxide, amines, sulfates, and simpler organic molecules.The suggested degradation pathway of AMX is depicted in Fig. 10.

### Cost estimation

The cost of photocatalytic treatment of 1 m^3^ of pharmaceutical wastewater is proportional to the quantity of waste per day and the volume of the reactor. As a cost guideline for the photocatalytic process, it is envisioned that a 10-m^3^ (Vc) reactor will be used in subsequent batches. Actual industrial wastewater usually differs from that treated in a laboratory due to the various matrix and competing pollutants, but such studies can fill the gap between research and practice of photocatalytic water treatment techniques, it will help. The study was calculated based on the optimal parameters obtained previously. The reactor is supposed to be a reinforced concrete structure with an age (*n*) of 25 years. One cycle time (*tc*) is 120 min, consisting of a reaction time for 1 cycle of 60 min and 60 min for reactor filling, emptying, ripening, and preparation. Three hundred days is the number of working days (*D*) in a year. Also, the number of working hours (*tw*) per day is 14 h. The basin volume (*CA*) was calculated using Eq. (16):

16 $${V}_{t} = CA\frac{D tw}{t\mathrm{c}}$$

where *V*_*t*_ is the volume of wastewater treated annually. The reactor capacity (*CA*) was determined to be 9.5 m^3^ but was roughly estimated to be 10.0 m^3^ to cover 5% of fluctuations in flow, based on a *V*_*t*_ of 20,000 m^3^. The total cost of the treatment process (*TC*) was estimated by considering both the amortization cost (*AC*) and the operating cost (*OC*). That is, *TC* = *AC* + *OC*. All the costs were equalized to 1 m^3^ of polluted wastewater. The AC cost was computed by summing up the costs of the basic constructing facilities and required equipment, including reinforced concrete body, air pump, mixer motor, and other necessary equipment. The *AC* per m^3^ of the industrial wastewater can be calculated by Eqs. (17–19) (Radwan et al. 2019):

17 $${AC}_{\mathrm{annual}}=C_{\mathrm p0}\left(\frac{\mathrm i{(1+\mathrm i)}^{\mathrm n}}{\left(1+\mathrm i\right)^{\mathrm n}-1}\right)$$

18 $${C}_{\mathrm{p}}={C}_{\mathrm{p}0}+n\times {AC}_{\mathrm{annual}}$$

19 $$AC=\frac{\mathrm{Cp}\times\mathrm{CA}}{\mathrm n\times\mathrm{Vt}}$$

where *C*_p0_ is the cost of constructing a photocatalytic reactor and permanent facilities, assessed to be 4200 $. *AC*_annual_ is the annual investment cost of *C*_p0_; *i* is the annual interest rate (7%); *C*_p_ is the net cost of the reactor volume. The *AC* for treating 1 m^3^ of polluted effluent is 0.26 $/m^3^. The *OC* is the cost of chemical materials, energy consumption, and maintenance. The cost of labor was not taken into account for simplifying the cost study. *C*_*ch*_ was estimated at 1.05 $/m^3^, including catalyst dose, potassium periodate, and pH adjustment if required. It was assumed that the cost of the required lamps (*C*_lamps_) would be 1.5 $. The energy consumed (*EC*) to operate the mixer, and pumps were determined in $ /m^3^ based on Eq. (20):

20 $$EC=\frac{\mathrm E\times\mathrm D\times\mathrm{PE}\times\mathrm{tw}}{\mathrm{Vt}}$$

where *E* is the energy consumed (kW); *PE* denotes the unit price of energy (0.18 $/kWh) (Radwan et al. 2019). The maintenance cost was assumed to be 2% of AC (Carra et al. 2013). Hence, the total *OC*, including consumables, energy, and maintenance, was estimated by Eq. (21).

21 $$OC={C}_{ch}+{C}_{\mathrm{lamps}}+EC+0.02AC$$

The average *EC* is 0.48 $/m^3^, so the *OC* is 3.04 $/m^3^, and the evaluated TC is 3.3 $/m^3^. The detailed costs were estimated and explained in Text S.1. It should be noted that these values may vary by location.

## Conclusions

The synthesis of ZnO nanocrystals by Zn-free MOFs is a novel approach to the use of MOFs in metal oxide processing. Zn based on MIL-53Al as a reactive template prepared via a simple hydrothermal operation was analyzed and applied as a photocatalyst for the degradation of AMX. The characterization of MIL-53(Al)/ZnO by a series of analyses confirmed a successful interaction between MIL-53Al and ZnO nanoparticles. The visible-light MIL-53(Al)/ZnO photocatalyst was first applied to remove AMX. The interaction model of the operating conditions was achieved by the RSM to maximize the AMX degradation by photoreaction after 60 min. The attained RSM model with *R*^2^ = 0.9569 exhibited a satisfying correlation between the expected and experimental values of AMX degradation. Superoxide radicals were the dominant active species in the photocatalytic quenching experiments. The reusability of suspended MIL-53(Al)/ZnO was verified in five continuous runs which revealed a highly stable photocatalytic activity after a prolonged period of reaction. In the presence of enhancers, the rate of AMX removal was accelerated and $${\mathrm{IO}}_{4}^{-}$$ exhibited a higher oxidizing power compared to the other oxidizing agents. The *COD* test has been performed to assess the elimination of AMX and the *COD* removal reached to 98.23% after 75 min of reaction. Moreover, the oxidation pathway was suggested with a thorough determination of the generated transformers by (LC–MS/MS) tandem mass spectroscopy. The cost estimation study displayed that treating 1 m^3^ of similar polluted water with AMX is 3.3 $/m^3^.

## Supplementary Information

Below is the link to the electronic supplementary material.

Supplementary file1 (DOCX 555 KB)

## Data Availability

All the data generated or analyzed during this study are included in this published article (and its supplementary information files).

## References

[CR1] Abazari R, Reza Mahjoub A, Slawin AMZ, Carpenter-Warren CL (2018). Morphology- and size-controlled synthesis of a metal-organic framework under ultrasound irradiation: an efficient carrier for pH responsive release of anti-cancer drugs and their applicability for adsorption of amoxicillin from aqueous solution. Ultrason Sonochem.

[CR2] Ani IJ, Akpan UG, Olutoye MA, Hameed BH (2018). Photocatalytic degradation of pollutants in petroleum refinery wastewater by TiO2- and ZnO-based photocatalysts: recent development. J Clean Prod.

[CR3] APHA, AWWA,WEF (2005) Standard methods for the examination of water and wastewater, 21st edn. APHA, Washington DC

[CR4] Arsand JB, Hoff RB, Jank L, Meirelles LN, Silvia Díaz-Cruz M, Pizzolato TM, Barceló D (2018). Transformation products of amoxicillin and ampicillin after photolysis in aqueous matrices: Identification and kinetics. Sci Total Environ.

[CR5] Camaratta R, Orozco Messana J, Pérez Bergmann C (2015). Synthesis of ZnO through biomimetization of eggshell membranes using different precursors and its characterization. Ceram Int.

[CR6] Carra I, Ortega-Gómez E, Santos-Juanes L, Casas López JL, Sánchez Pérez JA (2013). Cost analysis of different hydrogen peroxide supply strategies in the solar photo-Fenton process. Chem Eng J.

[CR7] Chaba JM, Nomngongo PN (2019). Effective adsorptive removal of amoxicillin from aqueous solutions and wastewater samples using zinc oxide coated carbon nanofiber composite. Emerg Contam.

[CR8] Chen L-J, Chuang Y-J (2012). Hydrothermal synthesis and characterization of hexagonal zinc oxide nanorods with a hexamethylenetetramine (HMTA) template-assisted at a low temperature. Mater Lett.

[CR9] Chen W-S, Jhou Y-C, Huang C-P (2014). Mineralization of dinitrotoluenes in industrial wastewater by electro-activated persulfate oxidation. Chem Eng J.

[CR10] Dehghan S, Kakavandi B, Kalantary RR (2018). Heterogeneous sonocatalytic degradation of amoxicillin using ZnO@Fe3O4 magnetic nanocomposite: influential factors, reusability and mechanisms. J Mol Liq.

[CR11] Dimitrakopoulou D, Rethemiotaki I, Frontistis Z, Xekoukoulotakis NP, Venieri D, Mantzavinos D (2012). Degradation, mineralization and antibiotic inactivation of amoxicillin by UV-A/TiO2 photocatalysis. J Environ Manage.

[CR12] El-Bendary N, El-Etriby HK, Mahanna H (2021). High performance removal of iron from aqueous solution using modified activated carbon prepared from corn cobs and luffa sponge. Desalin Water Treat.

[CR13] Elmolla ES, Chaudhuri M (2010). Photocatalytic degradation of amoxicillin, ampicillin and cloxacillin antibiotics in aqueous solution using UV/TiO2 and UV/H2O2/TiO2 photocatalysis. Desalination.

[CR14] Gar Alalm M, Samy M, Ookawara S, Ohno T (2018). Immobilization of S-TiO2 on reusable aluminum plates by polysiloxane for photocatalytic degradation of 2,4-dichlorophenol in water. J Water Process Eng.

[CR15] Grinnell C, and Samokhvalov AJPCCP (2018) Exploring the electronic structure of aluminum metal–organic framework Basolite A100: solid-state synchronous fluorescence spectroscopy reveals new charge excitation/relaxation pathways. 20(42): 26947–26956. 10.1039/C8CP04988B 10.1039/c8cp04988b 30310907

[CR16] Guha Ray P, Das M, Wan M, Jacob C, Roy S, Basak P, Dhara S (2020). Surfactant and catalyst free facile synthesis of Al-doped ZnO nanorods – an approach towards fabrication of single nanorod electrical devices. Appl Surf Sci.

[CR17] Han C, Yang M-Q, Weng B, and Xu Y-JJPCCP (2014) Improving the photocatalytic activity and anti-photocorrosion of semiconductor ZnO by coupling with versatile carbon. 16(32): 16891–16903. 10.1039/C4CP02189D 10.1039/c4cp02189d 25012572

[CR18] Hou D, Goei R, Wang X, Wang P, Lim T-T (2012). Preparation of carbon-sensitized and Fe–Er codoped TiO2 with response surface methodology for bisphenol A photocatalytic degradation under visible-light irradiation. Appl Catal B.

[CR19] Jalali S, Ardjmand M, Ramavandi B, Nosratinia F (2021). Removal of amoxicillin from wastewater in the presence of H2O2 using modified zeolite Y- MgO catalyst: An optimization study. Chemosphere.

[CR20] JonidiJafari A, Kakavandi B, Jaafarzadeh N, RezaeiKalantary R, Ahmadi M, Akbar Babaei A (2017). Fenton-like catalytic oxidation of tetracycline by AC@Fe3O4 as a heterogeneous persulfate activator: adsorption and degradation studies. J Ind Eng Chem.

[CR21] Jorfi S, Pourfadakari S, and Kakavandi BJCEJ (2018) A new approach in sono-photocatalytic degradation of recalcitrant textile wastewater using MgO@ Zeolite nanostructure under UVA irradiation. 343: 95–107. 10.1016/j.cej.2018.02.067

[CR22] Kanakaraju D, Kockler J, Motti CA, Glass BD, Oelgemöller M (2015). Titanium dioxide/zeolite integrated photocatalytic adsorbents for the degradation of amoxicillin. Appl Catal B.

[CR23] Khan H, Usen N, Boffito DC (2019). Spray-dried microporous Pt/TiO2 degrades 4-chlorophenol under UV and visible light. J Environ Chem Eng.

[CR24] Khataee A, Soltani RDC, Karimi A, Joo SW (2015). Sonocatalytic degradation of a textile dye over Gd-doped ZnO nanoparticles synthesized through sonochemical process. Ultrason Sonochem.

[CR25] Khataee A, Vahid B, Saadi S, Joo SW (2016). Development of an empirical kinetic model for sonocatalytic process using neodymium doped zinc oxide nanoparticles. Ultrason Sonochem.

[CR26] Ko F-H, Lo W-J, Chang Y-C, Guo J-Y, Chen C-M (2016). ZnO nanowires coated stainless steel meshes as hierarchical photocatalysts for catalytic photodegradation of four kinds of organic pollutants. J Alloy Compd.

[CR27] Kumar A, Kumar A, Sharma G, Al-Muhtaseb AAH, Naushad M, Ghfar AA, Stadler FJ (2018). Quaternary magnetic BiOCl/g-C3N4/Cu2O/Fe3O4 nano-junction for visible light and solar powered degradation of sulfamethoxazole from aqueous environment. Chem Eng J.

[CR28] Largani SH, and Pasha MAJINL (2017). The effect of concentration ratio and type of functional group on synthesis of CNT–ZnO hybrid nanomaterial by an in situ sol–gel process. **7**(1): 25–33.

[CR29] Leong KH, Liu SL, Sim LC, Saravanan P, Jang M, Ibrahim S (2015). Surface reconstruction of titania with g-C3N4 and Ag for promoting efficient electrons migration and enhanced visible light photocatalysis. Appl Surf Sci.

[CR30] Li X, Yu J, and Jaroniec MJCSR (2016) Hierarchical photocatalysts. 45(9): 2603–2636 10.1039/c5cs00838g 26963902

[CR31] Liu J, Zhao Y, Ma J, Dai Y, Li J, Zhang J (2016). Flower-like ZnO hollow microspheres on ceramic mesh substrate for photocatalytic reduction of Cr(VI) in tannery wastewater. Ceram Int.

[CR32] Mahanna H, Azab M (2020). Adsorption of reactive red 195 dye from industrial wastewater by dried soybean leaves modified with acetic acid. Desalin Water Treat.

[CR33] Martins AF, Mayer F, Confortin EC, Frank CdSJCS (2009). A study of photocatalytic processes involving the degradation of the organic load and amoxicillin in hospital wastewater. Air Water.

[CR34] Mirzaei A, Chen Z, Haghighat F, Yerushalmi L (2016). Removal of pharmaceuticals and endocrine disrupting compounds from water by zinc oxide-based photocatalytic degradation: a review. Sustain Cities Soc.

[CR35] Mirzaei A, Chen Z, Haghighat F, Yerushalmi L (2018). Hierarchical magnetic petal-like Fe3O4-ZnO@g-C3N4 for removal of sulfamethoxazole, suppression of photocorrosion, by-products identification and toxicity assessment. Chemosphere.

[CR36] Molla, MAI, Tateishi I, Furukawa M, Katsumata H, Suzuki T, and Kaneco SJOjoin-mm (2017). Evaluation of reaction mechanism for photocatalytic degradation of dye with self-sensitized TiO2 under visible light irradiation **7**(1): 1–7. 10.4236/ojinm.2017.71001

[CR37] Nägele E, Moritz R (2005). Structure elucidation of degradation products of the antibiotic amoxicillin with ion trap MSn and accurate mass determination by ESI TOF. J Am Soc Mass Spectrom.

[CR38] Neppolian B, Choi HC, Sakthivel S, Arabindoo B, Murugesan V (2002). Solar light induced and TiO2 assisted degradation of textile dye reactive blue 4. Chemosphere.

[CR39] Palma-Goyes RE, Vazquez-Arenas J, Ostos C, Ferraro F, Torres-Palma RA, Gonzalez I (2016). Microstructural and electrochemical analysis of Sb2O5 doped-Ti/RuO2-ZrO2 to yield active chlorine species for ciprofloxacin degradation. Electrochim Acta.

[CR40] Radwan M, Gar Alalm M, El-Etriby HK (2019). Application of electro-Fenton process for treatment of water contaminated with benzene, toluene, and p-xylene (BTX) using affordable electrodes. J Water Process Eng.

[CR41] Samy M, Ibrahim MG, Gar Alalm M, Fujii M (2020). Effective photocatalytic degradation of sulfamethazine by CNTs/LaVO4 in suspension and dip coating modes. Sep Purif Technol.

[CR42] Samy M, Ibrahim MG, Gar Alalm M, Fujii M (2020). MIL-53(Al)/ZnO coated plates with high photocatalytic activity for extended degradation of trimethoprim via novel photocatalytic reactor. Sep Purif Technol.

[CR43] Serna-Galvis EA, Silva-Agredo J, Giraldo-Aguirre AL, Flórez-Acosta OA, Torres-Palma RA (2016). High frequency ultrasound as a selective advanced oxidationprocess to remove penicillinic antibiotics and eliminate its antimicrobial activity from water. Ultrason Sonochem.

[CR44] Shayegan Z, Razzaghi M, Niaei A, Salari D, Tabar MTS, and Akbari ANJKJoCE (2013) Sulfur removal of gas oil using ultrasound-assisted catalytic oxidative process and study of its optimum conditions. 30(9): 1751–1759. 10.1007/s11814-013-0097-5

[CR45] Srikant V, and Clarke DRJJoAP (1998). On the optical band gap of zinc oxide. **83**(10): 5447–5451.

[CR46] Trovó AG, PupoNogueira RF, Agüera A, Fernandez-Alba AR, Malato S (2011). Degradation of the antibiotic amoxicillin by photo-Fenton process – chemical and toxicological assessment. Water Res.

[CR47] Xiao H, Zhang W, Yao Q, Huang L, Chen L, Boury B, Chen Z (2019). Zn-free MOFs like MIL-53(Al) and MIL-125(Ti) for the preparation of defect-rich, ultrafine ZnO nanosheets with high photocatalytic performance. Appl Catal B.

[CR48] Younes H, Mahanna H, El-Etriby HK (2019). Fast adsorption of phosphate (PO4−) from wastewater using glauconite. Water Sci Technol.

[CR49] Zhao D, Yu Y, and Chen JPJRa (2016) Fabrication and testing of zirconium-based nanoparticle-doped activated carbon fiber for enhanced arsenic removal in water. 6(32): 27020–27030. 10.1039/C5RA25030G.

[CR50] Zhao F, Liu Y, Hammouda SB, Doshi B, Guijarro N, Min X, Tang C-J, Sillanpää M, Sivula K, Wang S (2020). MIL-101(Fe)/g-C3N4 for enhanced visible-light-driven photocatalysis toward simultaneous reduction of Cr(VI) and oxidation of bisphenol A in aqueous media. Appl Catal B.

[CR51] Zhou L, Wang W, Xu H, Sun S, and Shang MJCAEJ (2009) Bi2O3 hierarchical nanostructures: controllable synthesis, growth mechanism, and their application in photocatalysis. 15(7): 1776–1782. 10.1002/chem.200801234 10.1002/chem.200801234 19115297

