# Supplementary material for: Effective photocatalytic degradation of amoxicillin using MIL-53(Al)/ZnO composite
Source: Environ Sci Pollut Res Int. 2022 May 11;29(45):68532–46. doi: 10.1007/s11356-022-20527-0 (PMC9508224; doi:10.1007/s11356-022-20527-0)
Supplement: Supplementary file 1 — Supplementary file1 (DOCX 555 KB) [file 11356_2022_20527_MOESM1_ESM.docx]

**Supplementary Material**

**Effective photocatalytic degradation of amoxicillin using MIL-53(Al)/ZnO composite**

**Asmaa Fawzy^a,b^ ,Hani Mahanna^a,*^,Mohamed Mossad^a,c^**

^a^ Public Works Engineering Department, Faculty of Engineering, Mansoura University, Mansoura, 35516, Egypt

^b^ E-mail address: [asmaafawzy529@gmail.com](mailto:asmaafawzy529@gmail.com)

^c^ E-mail address: [Maahm@mans.edu.eg](mailto:Maahm@mans.edu.eg)

^*^ Corresponding author. E-mail address: [Hany_mss@mans.edu.eg](mailto:Hany_mss@mans.edu.eg) (Hani Mahanna)

**Table S.1.** Determination of the limit of detection.

| No. | HPLC area | | Calculated concentration |
| --- | --- | --- | --- |
| 1 | 59.96 | 0.409035083 | |
| 2 | 60.91 | 0.578131397 | |
| 3 | 62.32 | 0.829105925 | |
| 4 | 62.92 | 0.935903597 | |
| 5 | 64.88 | 1.284775992 | |
| 6 | 69.89 | 2.176536552 | |
| 7 | 70.14 | 2.221035581 | |
| 8 | 70.23 | 2.237055232 | |
| 9 | 85.77 | 5.003114932 | |
| 10 | 88.37 | 5.465904843 | |
| 11 | 97.13 | 7.025150852 | |
| 12 | 97.28 | 7.05185027 | |
| 13 | 97.41 | 7.074989765 | |
| 14 | 102.06 | 7.902671722 | |
| 15 | 113.63 | 9.962086827 | |
| 16 | 121.87 | 11.42877485 | |
| 17 | 129.33 | 12.75662591 | |
| 18 | 225.39 | 30 | |

SE (Standard error of intercept) = 0.035051477

SD (Standard deviation) = SE * $\sqrt{N}$ =0.035051477 * $\sqrt{18}$= 0.148710823

LOD (Limit of detection) = 3.3(SD/slope) = 0.087350833 mg/L

**Fig.S.1.** Determination of point of zero charge MIL-53ZnO.


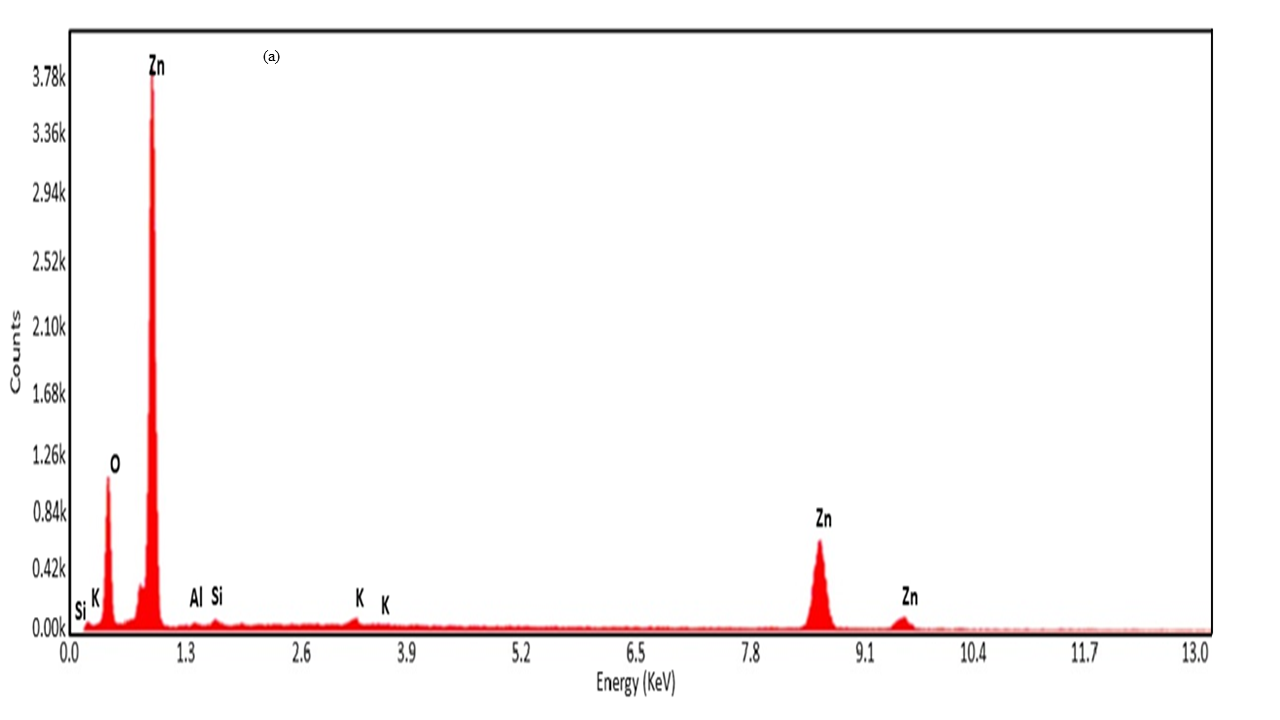


(b)

**Fig.S.2**(a) EDX pattern of MIL-53(Al)/ZnO; (b) FTIR spectra of MIL-53(Al)/ZnO.

**Table S.2**

The catalyst composition by weight% of each component.

| Weight % | Element |
| --- | --- |
| 28.97 | Ok |
| 1.24 | Al K |
| 1.26 | Si K |
| 68.53 | Zn K |

**Table S.3**

Experimental design conditions, the actual and the predicted values of AMX removal efficiency.

| Run | Coded values of parameters | | | Measured values of parameters | | | Y (AMX Removal ratio %) | |
| --- | --- | --- | --- | --- | --- | --- | --- | --- |
|  | X1 | X2 | X3 | X1 | X2 | X3 | Actual | Predicted |
| 1 | 1 | 1 | 1 | 9 | 0.8 | 70 | 61.49 | 58.793 |
| 2 | -1 | -1 | -1 | 5 | 0.4 | 30 | 88.49 | 90.666 |
| 3 | 0 | -2 | 0 | 7 | 0.2 | 50 | 74.94 | 69.258 |
| 4 | -1 | -1 | 1 | 5 | 0.4 | 70 | 67.73 | 81.271 |
| 5 | 0 | 0 | 2 | 7 | 0.6 | 90 | 59.89 | 62.71 |
| 6 | 0 | 0 | 0 | 7 | 0.6 | 50 | 80.14 | 79.648 |
| 7 | 0 | 0 | 0 | 7 | 0.6 | 50 | 78.72 | 79.648 |
| 8 | 0 | 0 | 0 | 7 | 0.6 | 50 | 78.19 | 79.648 |
| 9 | 0 | 0 | 0 | 7 | 0.6 | 50 | 81.02 | 79.648 |
| 10 | 0 | 2 | 0 | 7 | 1 | 50 | 77.95 | 84.153 |
| 11 | 2 | 0 | 0 | 11 | 0.6 | 50 | 43.63 | 42.095 |
| 12 | 1 | -1 | 1 | 9 | 0.4 | 70 | 49.89 | 51.301 |
| 13 | 1 | 1 | -1 | 9 | 0.8 | 30 | 70.05 | 68.078 |
| 14 | 0 | 0 | -2 | 7 | 0.6 | 10 | 83.69 | 81.39 |
| 15 | 1 | -1 | -1 | 9 | 0.4 | 30 | 56.54 | 62.346 |
| 16 | -1 | 1 | -1 | 5 | 0.8 | 30 | 100 | 98.068 |
| 17 | -1 | 1 | 1 | 5 | 0.8 | 70 | 96.76 | 90.433 |
| 18 | -2 | 0 | 0 | 3 | 0.6 | 50 | 100 | 102.055 |

**Actual:** AMX removal determined by experimental work.

**Predicted:** AMX removal calculated by Eq. (5) using Minitab V.19 software using RSM

**Table.S.4**

ANOVA test for the removal efficiency of AMX.

| Term | Degree of freedom | Sum of squares | Mean square | F-Value | P-Value |
| --- | --- | --- | --- | --- | --- |
| Model | 9 | 4290.17 | 476.69 | 19.74 | 0 |
| Linear | 3 | 4166.01 | 1388.67 | 57.52 | 0 |
| pH | 1 | 3595.2 | 3595.2 | 148.91 | 0 |
| Catalyst dose | 1 | 221.86 | 221.86 | 9.19 | 0.016 |
| Concentration | 1 | 348.94 | 348.94 | 14.45 | 0.005 |
| Square | 3 | 119.86 | 39.95 | 1.65 | 0.253 |
| pH*pH | 1 | 78.19 | 78.19 | 3.24 | 0.11 |
| Catalyst dose*Catalyst dose | 1 | 11.81 | 11.81 | 0.49 | 0.504 |
| Concentration*Concentration | 1 | 78.71 | 78.71 | 3.26 | 0.109 |
| Two-way interaction | 3 | 4.3 | 1.43 | 0.06 | 0.98 |
| pH*Catalyst dose | 1 | 1.39 | 1.39 | 0.06 | 0.816 |
| pH*Concentration | 1 | 1.36 | 1.36 | 0.06 | 0.818 |
| Catalyst dose*Concentration | 1 | 1.55 | 1.55 | 0.06 | 0.806 |
| Error | 8 | 193.15 |  |  |  |
| Lack-of-Fit | 5 | 188.11 |  | 22.38 | 0.014 |
| Pure Error | 3 | 5.04 |  |  |  |
| Total | 17 | 4483.32 |  |  |  |

**R^2^** = 95.69 %, **R^2^ (adj)** = 90.85 %

**Table.S.5**

Comparison of reported mineralization and removal efficiencies of AMX by various photocatalysts.

| Photo catalyst | Experimental conditions | | | Reported results | | References |
| --- | --- | --- | --- | --- | --- | --- |
|  | Initial conc. (mg/L) | pH | Catalyst dose (g/L) | Removal % | Time (hr) |  |
| TiO_2_ | 30 | 7 | 0.45 | 80 | 4.5 | (Verma and Haritash,2020) |
| Mesoporous g-C_3_N_4_ | 1 | 7 | 0.1 | 99 | 1 | (Dou, Wang et al.,2020) |
| Metal-free carbon Nitride | 0.5 | 7 | 0.03 | 100 | 48 | (Silva, Teixeira et al.,2021) |
| Hetrojunction p-CuO/n-ZnO | 50 | 11 | 0.5 | 93 | 4 | (Belaissa, Nibou et al.,2016) |
| MIL-68(In)-NH2/GrO composite | 20 | 5 | 0.6 | 93 | 2 | (Yang, You et al.,2017) |
| MIL-53(Al)/ZnO | 10 | 4.5 | 1 | 100 | 1 | This study |

**Table.S.6**

Bandgap parameters for single photo catalysts.

| Photocatalyst | X | E_VB_ | E_CB_ | E_g_ |
| --- | --- | --- | --- | --- |
| ZnO | 6 | 3.15 | -0.15 | 3.3 |
| MIL-53Al | 6.56 | 3.81 | 0.31 | 3.5 |

**Fig. S.3** Influence of different oxidizing agents on the photocatalytic degradation of AMX by MIL-53(Al)/ZnO/UV system (initial AMX con.=10 mg/L, pH=4.5, oxidant con. = 2 mM).


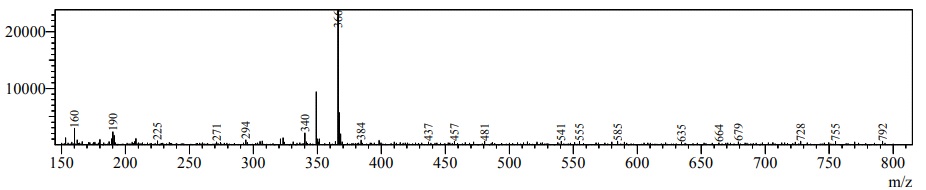

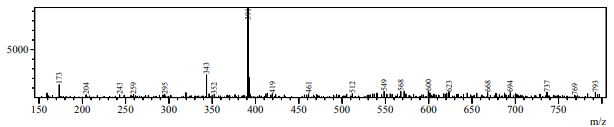


(b)

(a)

(a)


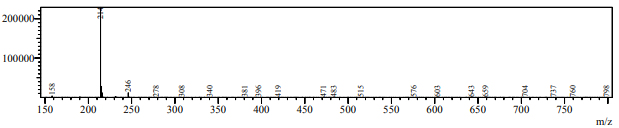


(c)


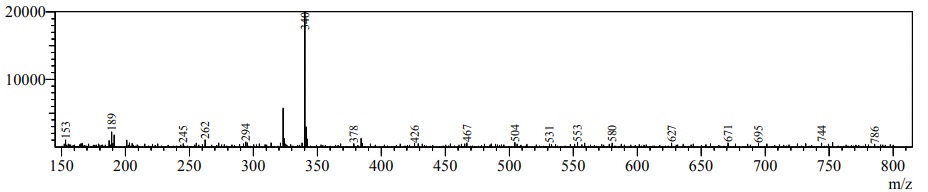


(d)


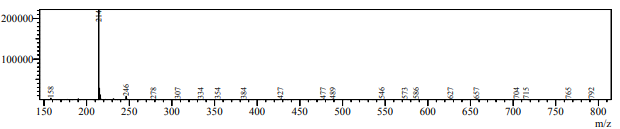

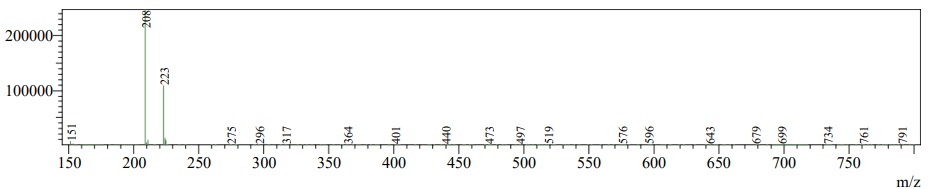

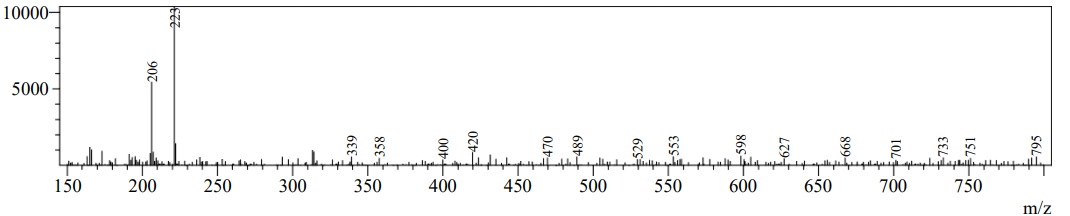

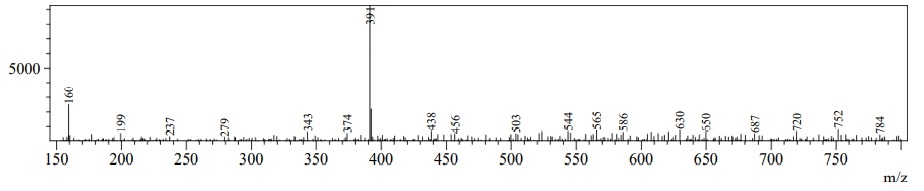


(f)

(e)

(g)

(h)


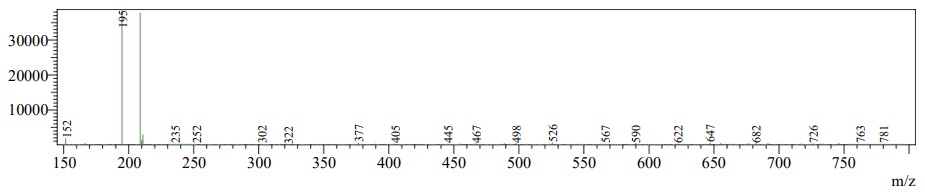


(i)

**Fig.S.4**. LC-MS/MS intermediate transformation byproducts formed during the photo degradation of AMX, (a-c) represent Untreated sample, (e-i) represent final treated sample at end of experimental run.

**Table.S.6**. The residence times obtained.

| m/z | R. time (min) |
| --- | --- |
| 366 | 3.27 |
| 340 | 3.57 |
| 391 | 26.6 |
| 214 | 15.47 |
| 223 | 27.68 |
| 206 | 29.08 |
| 208 | 29.42 |
| 195 | 28.78 |

**Text S1: Cost calculations**

The dimensions of a cylindrical full-scale photocatalytic reactor are a dimeter =2.0 m, height = 3.0 m, wall thickness = 0.25 m, and floor thickness = 0.3 m. Accordingly, the total volume of reinforced concrete is 6.8 m^3^. The cost of total reinforced concrete was estimated based on the use of 350 kg/m^3^ of cement and 120 kg/m^3^ of steel including the cost of required equipment and labor to be 1500 $. The cost of the DC power supply including the required accessories was 1000 $ and the cost of the mixer was 1000 $ according to our investigation. The cost of the air pump was 500 $ and the costs of the required connections for the operation were assumed to be 100 $. Therefore, the cost of the reactor construction and permanent facilities (C_m0_) was roughly calculated to be 4200 $.

The cost of preparation of one kg of the catalyst was determined to be 2.3 $ including the costs of the precursors and chemicals. The dose of the catalyst was determined to be 0.2 kg/m^3^. The cost of one kg of Potassium periodate is about 2 $. A Potassium periodate dose of 0.12 kg /m^3^ was considered for the calculation. For the pH adjustment, the costs of HCl and NaOH were determined to be 0.04 $/ L. and 0.03 $/kg, respectively. Accordingly, the total cost of chemicals (C_ch_) was 1.05 $/m^3^. The number of lamps needed in large-scale reactor to attain the same illumination intensity in lab-scale reactor is considered to be 12 lamps with a power of 400 W. assume the cost of lamps needed to illuminate one cubic meter of wastewater is 1.5 $ Moreover, the power required for the operation of pumps, lamps, and mixers was assumed to be 12.6 KW. Accordingly, the total cost of energy (EC) including mixers, pumps, lamps and catalyst preparation was calculated to be 0.48 $/m^3^.
